# Genetic diversity and population structure among six cattle breeds in South Africa using a whole genome SNP panel

**DOI:** 10.3389/fgene.2014.00333

**Published:** 2014-09-22

**Authors:** Sithembile O. Makina, Farai C. Muchadeyi, Este van Marle-Köster, Michael D. MacNeil, Azwihangwisi Maiwashe

**Affiliations:** ^1^Agricultural Research Council-Animal Production InstituteIrene, South Africa; ^2^Department of Animal and Wildlife Sciences, University of PretoriaHatfield, South Africa; ^3^Agricultural Research Council-Biotechnology PlatformOnderstepoort, South Africa; ^4^Department of Animal, Wildlife and Grassland Sciences, University of Free StateBloemfontein, South Africa; ^5^Delta G, Miles CityMT, USA

**Keywords:** South Africa, cattle breeds, genetic resources, genetic diversity, population structure

## Abstract

Information about genetic diversity and population structure among cattle breeds is essential for genetic improvement, understanding of environmental adaptation as well as utilization and conservation of cattle breeds. This study investigated genetic diversity and the population structure among six cattle breeds in South African (SA) including Afrikaner (*n* = 44), Nguni (*n* = 54), Drakensberger (*n* = 47), Bonsmara (*n* = 44), Angus (*n* = 31), and Holstein (*n* = 29). Genetic diversity within cattle breeds was analyzed using three measures of genetic diversity namely allelic richness (A_R_), expected heterozygosity (H_e_) and inbreeding coefficient (*f*). Genetic distances between breed pairs were evaluated using Nei's genetic distance. Population structure was assessed using model-based clustering (ADMIXTURE). Results of this study revealed that the allelic richness ranged from 1.88 (Afrikaner) to 1.73 (Nguni). Afrikaner cattle had the lowest level of genetic diversity (H_e_ = 0.24) and the Drakensberger cattle (H_e_ = 0.30) had the highest level of genetic variation among indigenous and locally-developed cattle breeds. The level of inbreeding was lower across the studied cattle breeds. As expected the average genetic distance was the greatest between indigenous cattle breeds and *Bos taurus* cattle breeds but the lowest among indigenous and locally-developed breeds. Model-based clustering revealed some level of admixture among indigenous and locally-developed breeds and supported the clustering of the breeds according to their history of origin. The results of this study provided useful insight regarding genetic structure of SA cattle breeds.

## Background

African cattle breeds can be divided into two major categories, namely Taurine cattle (*Bos taurus*) and Indicine cattle (*Bos indicus*). *Bos indicus* is subdivided into zebu proper and zebu crossbred-types and is phenotypically identifiable by the presence of a substantial cerciothoracic hump (Rege, 1999). The position of the hump on the animal's back is used to classify the zebu proper and zebu crossbred types into cervico thoracic-humped and thoracic-humped stocks (Epstein, 1971). Cervico-thoracic-humped cattle occur in or are derived from, contact areas of thoracic-humped Zebu and humpless cattle. In crossbreds of humped and thoracic-humped Zebu cattle, the hump is usually cervico-thoracic and these cattle are referred to as Sanga. However, the Sanga is nowadays considered a separate group of cattle. Thus, African cattle can be classified into four different groups distinguished namely *B. taurus, B. indicus, Sanga*, and *Sanga' zebu types* (Rege, 1999). Afrikaner and Nguni cattle are classified under the Sanga group and indigenous to South Africa. Drakensberger and Bonsmara cattle are also classified under Sanga types, however, the origin of the Drakensberger cattle is unclear with a history dating back to the early settlers in the late 1700's (Scholtz et al., 2010). The Bonsmara cattle was developed at Mara and Messina Research Station from 1937 to 1963 using Milk Short Horn, Hereford, and Afrikaner cattle with the aim to produce a locally adapted beef breed (Bonsma, 1980). Angus and Holstein belong to *Bos taurus* group and these originate from British and Europe, respectively.

The Afrikaner is one of the oldest breeds with a medium–frame, yellow to red colored with lateral horns with a typical twist. It has exceptional good quality meat and is the ideal minimum care and maximum profit breed (Strydom et al., 2000). Nguni cattle are characterized by their multi-colored coats, which can present many different patterns (white, brown, golden yellow, black, dappled, or spotty), but their noses are always black-tipped and they present a variety of horn shapes. This small framed breed has been kept in rural areas for centuries and often used as dam lines in crossbreeding systems (Scholtz et al., 2011). Drakensberger is a medium to large frame breed and has a black smooth coat. A study by Strydom (2008) has shown that the Drakensberger compare well to British and Europe breeds with regard to meat quality. Bonsmara is medium to large framed, smooth coated with heat and tick tolerance and current the breed with the largest number of registered females in South Africa (Muchenje et al., 2008).

*Bos indicus* are known to be adapted to the sub-tropical areas in Africa and have a higher tolerance to various diseases (Muchenje et al., 2008; Marufu et al., 2011). These breeds are also suited to low input systems with lower maintenance and management requirements. In a changing South African environment breeds such as the Afrikaner, Nguni, Drakensberger, and Bonsmara holds potential. Despite their large numbers and not endangered, breeds genetic diversity information is essential for control of inbreeding and effective utilization of breed specific characteristics. The adaptive traits are of importance and there is worldwide a drive for effective management of indigenous genetic resources as they could be most valuable in selection and breeding programs in times of biological stress such as famine, drought, or disease epidemics (FAO, 2010). In order to effectively manage these cattle breeds comprehensive knowledge of their characteristics is required. These include population size and structure as well as knowledge of within and between breeds' divergence (Boettcher et al., 2010; Groeneveld et al., 2010). In South Africa a number of studies have focused on the characterization of small stock such as goats: Visser et al. (2004); sheep: Soma et al. (2012), Qwabe et al. (2012). Limited studies have focused on the genetic characterization of South African cattle breeds and this thus emphasized the need for a genetic characterization of these breeds as genetic resources.

Worldwide genetic markers have been used to assess the genetic variation among many cattle breeds relative to their area of origin (Blott et al., 1998; Hanotte et al., 2002; Gautier et al., 2007; Edea et al., 2013). Results have shown that genetic diversity of breeds is directly linked to their areas of origin, indicating that breeds which have diverged more recently were generally closer together geographically. These studies have also demonstrated larger differences between taurine and indicine breeds due to a greater time since their divergence (McKay et al., 2008; Edea et al., 2013). In addition, significant differences were reported between beef and dairy cattle compared to within beef or dairy; this was attributed to different selection pressure across these contemporary groups (Hayes et al., 2003).

This study therefore investigated genetic diversity and population structure within and between six cattle breeds in South African including Afrikaner, Nguni, Drakensberger, Bonsmara, Angus, and Holstein using genome wide single nucleotide polymorphism (SNP) generated from the Illumina Bovine SNP50BeadChip.

## Materials and methods

### Animal resources

**Figure 1:**
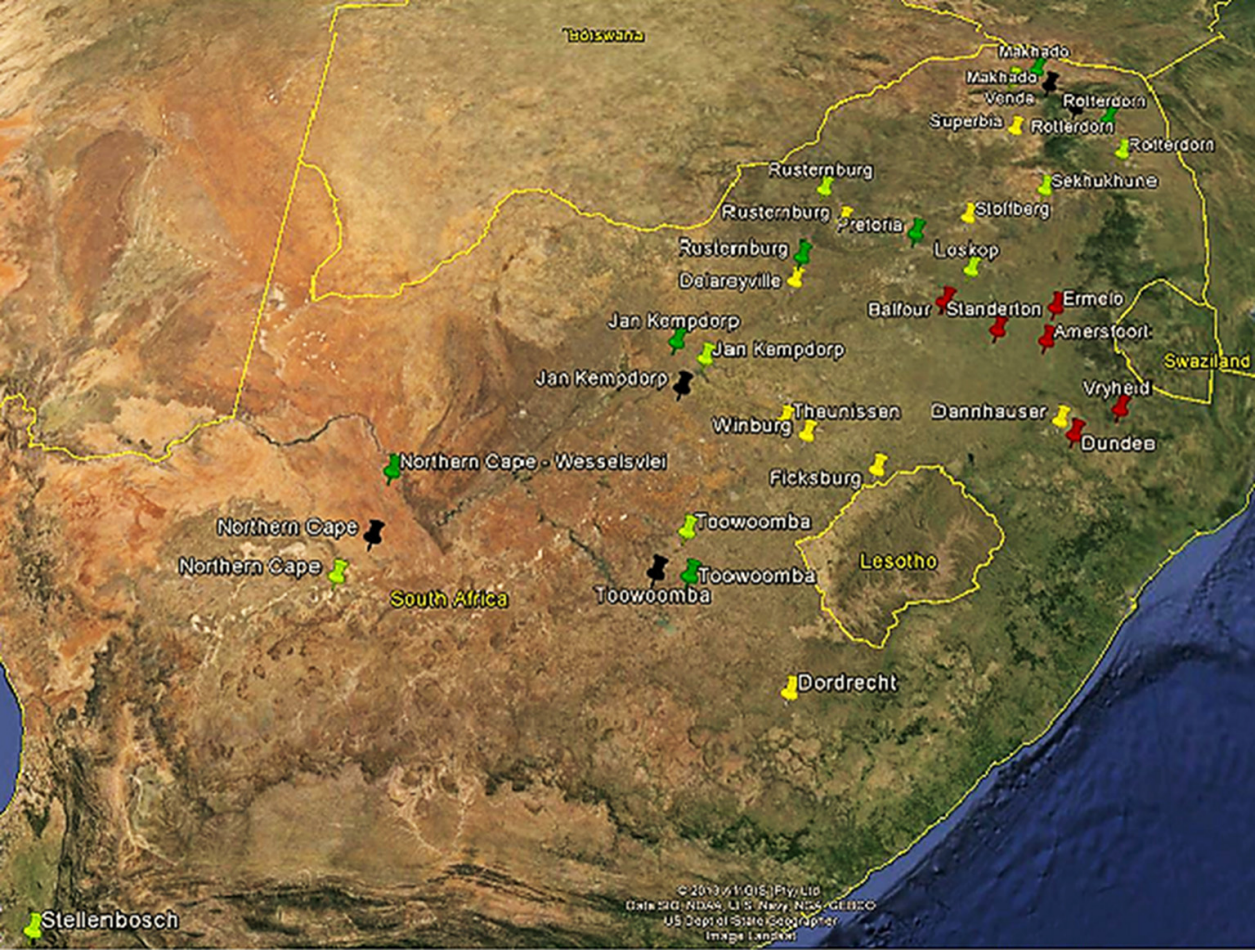
**Geographic origin of five cattle breeds in South Africa sampled in the current study**. Afrikaner (yellow) (44), Nguni (light green) (56), Drakensbureger (red) (47), Bonsmara (dark green), and Angus (black) (31).

A total of 249 animals including three indigenous breeds (Afrikaner = 44), (Nguni = 54), (Drakensberger = 47), one composite (locally-developed) (Bonsmara = 44), and two *Bos taurus* (Angus = 31) and (Holstein = 29) cattle breeds were included in this study. Breeders and Research Stations which keep pure breeds of the populations included in this study were identified and requested to provide animals for blood sampling. All animal handling and sample collection were done according to the regulations of the Animal Ethics Committee of the University of Pretoria (E087-12). To maximize the genetic diversity within each sampled population, pedigree data were used to select against full and half sib animals. Figure 1 show the map of South Africa indicating the location of farms and research station where populations under study were sampled. The sampling of these animals included collection of 10 ml whole blood using EDTA VACUETTE® tubes. Holstein (48) semen samples were obtained with permission from an artificial insemination company (Taurus, South Africa). However, to maximize the genetic diversity within Holstein samples, identity by descent analysis was performed using data generated from the Bovine SNP50 BeadChip to select the least related bulls. In which a total of 29 least related bulls were selected for the purpose of this study.

### Genotyping and quality control

Genomic DNA was extracted at the ARC-Biotechnology Platform from whole blood and semen samples using the Qiagen DNeasy extraction kit (Qiagen, South Africa) according to the manufacturer's protocol. The protocol was adapted for the semen samples where Dithiothreitol (DTT) was added with proteinase K in the first step. Genomic DNA for all samples was quantified using a Qubit® 2.0 Fluorometer and the Nanodrop Spectrophotometer (Nanodrop ND-1000). In addition, gel electrophoresis was performed to quantify the DNA.

Genotyping was conducted at the ARC-Biotechnology Platform with the Illumina BovineSNP50 BeadChip v2 which features 54,609 SNP probes distributed across the whole bovine genome with an average spacing of 49.9 kb (Matukumalli et al., 2009). Approximately 12 μL of DNA loaded in each well of a BeadChip of genomic DNA was used to genotype each sample. Samples were processed according to the Illumina Infinium–II assay protocol (Illumina, Inc. San Diego, CA, 92122, USA). Quality control criteria were performed across six cattle breeds to remove from further analysis any SNPs with less than 95% call rate, SNPs with less than 0.02 MAF and samples with more than 10% missing genotypes (Purcell et al., 2007). This left about 46,236 SNPs across the breeds. Furthermore, SNPs that were in high LD were pruned using the following parameter; –indep 50 5 2 in plink (Purcell et al., 2007); this left about 21,290 SNPs for further analysis. Pruning of SNPs that are in high LD have been shown to counter the effect of ascertainments bias and to generate meaningful comparison between breeds (Kijas et al., 2009).

### Estimates of within breed genetic diversity

Three measures of genetic variability were used to compare the levels of heterogeneity within the cattle breeds (allelic richness, expected heterozygosity, and inbreeding coefficient). Allelic richness (A_R_) was determine within each population using ADZE v 1.07 (Szpiech et al., 2008), while expected heterozygosity (H_e_) and Inbreeding coefficient (*f*) was calculated using Plink v1.07 (Purcell et al., 2007) under the default setting.

### Analyses of molecular variance (AMOVA) and population differentiation

Analyses of molecular variance to determine the partition of genetic diversity was first performed among indigenous and locally-developed cattle breeds and then amongst all six cattle breeds with the program ARLEQUIN 3.1 version (Excoffier et al., 2005).

Populations differentiation was evaluated using pairwise *F_ST_* estimates according to Weir and Cockerham (1984) using Golden Helix SNP Variation Suite (SVS) Version 8.1(Golden Helix Inc., Bozeman, MT, 2012).

### Allele sharing and genetic distance

Genetic distance between all pairwise combination of individuals (*D*) was estimated as one minus the average proportion of allele shared (Purcell et al., 2007) where the average proportion of allele shared was calculated as Dst using Plink v1.07 (Purcell et al., 2007) as:

$$\text{Dst}=\frac{\text{IBS2}+0.5^{*}\text{IBS1}}{\text{N}}$$

Where IBS1 and IBS2 are the number of loci which are shared either 1 or 2 alleles identical by state (IBS), respectively, and N is the number of loci tested.

Pairwise genetic distance among cattle breeds was estimated based on Nei's(1987) unbiased genetic distance using Phylip v 3.695 genetic software (Felsenstein, 1989), in which a Neighbor-joining (NJ) relationship tree was then constructed using DrawTree application within Phylip v 3.695 software (Felsenstein, 1989).

### Structure analysis

**Figure 2:**
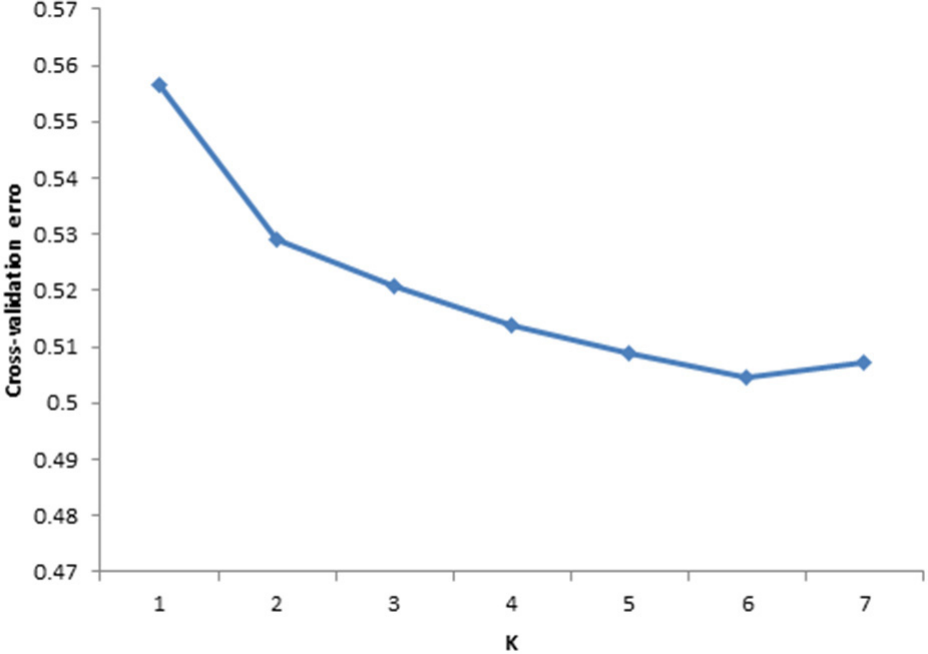
**Cross validation plot for six cattle breeds in South Africa**. Based on cross validation error the plot indicated that *k* = 6 is optimal for data set.

To investigate the population structure of the studied cattle breeds, ADMIXTURE 1.2.3 Software (Alexander et al., 2009) was used. In order to infer the true number of genetic populations (clusters or K) between the six cattle breeds. Prior population information was ignored before testing and identifying distinct genetic populations, and assigning individuals to populations. ADMIXTURE uses cross validation (CV) procedure to estimate most preferable *K*. Most preferable *K* exhibit a low cross-validation error compared to other *K*-values. In the current study CV error estimates were plotted (Figure 2) for comparison of *K* and *K* = 6 exhibited low cross validation error values thus *K* = 6 was taken as the most probable number of inferred populations.

## Results

### SNP polymorphism and within breed genetic diversity

Parameter for SNP validation that included the level of polymorphism, minor allele frequency (MAF) and deviation from Hardy Weinberg equilibrium (HWE) for all six cattle breeds in this study were previously reported (Makina et al., submitted). In summary, examination across breeds revealed that about 56% of SNPs were polymorphic in all breeds and the distribution of MAF showed that nearly half of the SNPs (41%) showed a higher degree of polymorphism (MAF ≥ 0.05) across the breeds. With regard to deviation from HWE only between 5 and 6% of SNP were shown to deviate from HWE (*P* ≤ 0.05) across the six breeds.

Table 1 presents three measures of within breed diversity across the breeds: Afrikaner cattle had the highest number alleles per locus (A_R_ = 1.88) while the Nguni cattle had the lowest number of alleles per locus (A_R_ = 1.73). However, the Afrikaner cattle was observed to have the lowest level of expected heterozygosity (H_e_ = 0.24) in this study. Among indigenous and locally-developed breeds the Drakensberg cattle (H_e_ = 0.30) had the highest level of genetic diversity. Looking across all six breeds Angus and Holstein cattle had the highest level of gene diversity (H_e_ = 0.31). The level of inbreeding was low across the breeds in this study ranging from 0.004 (Afrikaner) to −0.002 (Drakensberger).

**Table 1:** **Sample size and genetic diversity within six cattle breeds in South Africa**.

**Breed**	**Code**	***n***	**A**_R_** (***SD***)**	**H**_e_** (*SD*)**	****F_is_****
Afrikaner	AFR	42	1.88 (0.12)	0.24 (0.18)	0.004
Nguni	NGU	54	1.73 (0.11)	0.28 (0.17)	0.005
Drakensberger	DRA	47	1.85 (0.12)	0.30 (0.17)	−0.002
Bonsmara	BON	44	1.84 (0.11)	0.29 (016)	−0.017
Angus	ANG	31	1.80 (0.13)	0.31 (0.16)	−0.012
Holstein	HOL	29	1.81 (0.13)	0.31 (0.18)	−0.026

### Analyses of molecular variance and population differentiation

Analysis of Molecular Variance illustrated that within breed genetic variation accounted for 90% among indigenous and locally-developed breeds. On the other hand when indigenous and locally-developed breeds were grouped together with *Bos taurus* cattle 92% of genetic diversity occurred within breeds while only 8% occurred between the breeds (Table 2).

**Table 2:** **Analysis of Molecular Variance among six cattle breeds in South Africa**.

**Data set**	**Variance component (%)**		
	**Among groups**	**Among populations within group**	**Within populations**
All six cattle breeds	7.80	0.70	91.45
Indigenous and local developed breeds	7.80	1.40	90.80

Populations differentiation estimates showed that *F_ST_* varied from 0.043 (Nguni-Drakensberger) to 0.081 (Afrikaner-Drakensberger) among indigenous and locally-developed breeds and from 0.078 (Drakensberger-Angus) to 0.159 (Afrikaner-Holstein) across all six breeds (Table 3).

**Table 3:** **Wright fixation index (*F_ST_*) pair-wise among six cattle breeds in South African**.

	**Afrikaner**	**Nguni**	**Drakensberger**	**Bonsmara**	**Angus**	**Holstein**
Afrikaner						
Nguni	0.064					
Drakensberger	0.080	0.044				
Bonsmara	0.071	0.044	0.043			
Angus	0.151	0.108	0.078	0.083		
Holstein	0.159	0.114	0.084	0.099	0.098	

### Genetic distance within and between cattle breeds

The average genetic distance between individuals drawn from the same breeds was 0.20 ± 0.01 within the Afrikaner cattle, 0.23 ± 0.01 within the Nguni, 0.25 ± 0.01 with the Drakensberger, 0.24 ± 0.01 within the Bonsmara, 0.25 ± 0.02 within the Angus and Holstein 0.25 ± 0.01. The average genetic distance between individuals drawn from different breeds ranged from 0.23 ± 0.005 (Afrikaner-Nguni) to 0.29 ± 0.004 (Angus and Holstein).

**Figure 3:**
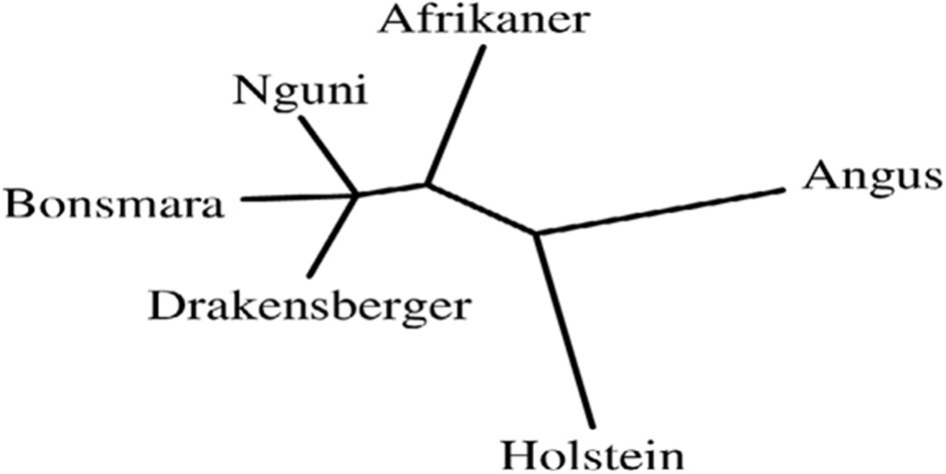
**Genetic distances between six cattle breeds in South Africa: Neighbor-joining relationship tree of tested cattle breeds**.

Topological relationships between breeds, from Neighbor-Joining tree clearly separated *Bos taurus* breeds (Angus and Holstein) from indigenous and locally-developed cattle breeds (Afrikaner, Nguni, Drakensberger, and Bonsmara) (Figure 3). Three main groups were separated: the group formed by Nguni, Drakensberger, and Bonsmara, the group formed by Afrikaner cattle and the group formed by the *Bos taurus* breeds (Angus and Holstein).

### Population structure analysis between six cattle breeds in south africa

**Figure 4:**
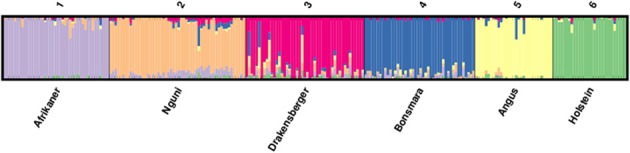
**ADMIXTURE clustering of six cattle breeds in South Africa**.

The proportions of individuals in each of the breeds in the six most likely clusters inferred by the ADMIXTURE are presented in Table 4 and this corresponded to the six different breeds included in the study. This revealed that 94% of Afrikaner breed were assigned to cluster one, 84% of Nguni were assigned to cluster two with 8% of its genome assigned to cluster one, 81% of Drakensberger were assigned to cluster three with 5% of its genome assigned to clusters two, four, and five, 89% of Bonsmara were assigned to cluster four with 3% of its genome assigned to cluster two, 93% of Angus were assigned to cluster five and 97% of Holstein were assigned to cluster six. The results presented in Figure 4 (*k* = 6) demonstrated that among the SA indigenous and locally-developed breeds (Afrikaner, Nguni, Drakensberger, and Bonsmara), the Afrikaner population had the least level of admixture while the Drakensberger had the most level of admixture. The Nguni cattle showed some signals of admixture with Afrikaner breed while the Drakensberger cattle revealed some signals of admixture with Nguni, Bonsmara, and Angus. Bonsmara cattle shared more genetic links with the Nguni cattle than with other indigenous breeds. When comparing all six breeds Afrikaner, Angus, and Holstein populations showed the lowest level of admixture in the current study.

**Table 4:** **Proportion of membership of the analyzed South African cattle breeds in each of the six clusters inferred in the ADMIXTURE program**.

**Predefined populations**	**Inferred clusters**						
	**1**	**2**	**3**	**4**	**5**	**6**	***n***
Afrikaner	**0.938**	0.036	0.011	0.006	0.005	0.011	42
Nguni	0.083	**0.838**	0.032	0.032	0.007	0.009	54
Drakensberger	0.032	0.048	**0.806**	0.040	0.045	0.028	47
Bonsmara	0.005	0.030	0.013	**0.887**	0.017	0.006	44
Angus	0.003	0.012	0.005	0.034	**0.932**	0.015	31
Holstein	0.000	0.000	0.008	0.005	0.018	**0.969**	29

*Bold indicate inferred cluster*.

## Discussion

Information about genetic diversity and population structure among cattle breeds is essential for genetic improvement, understanding of environmental adaptation as well as utilization and conservation of cattle breeds (Groeneveld et al., 2010). This study investigated the genetic diversity and population structure among six cattle breeds in South Africa. Among indigenous and locally-developed breeds; Drakensberger cattle demonstrated the highest level of genetic variability (H_e_ = 0.30) while the Afrikaner demonstrated the lowest level of genetic diversity. The lower level of genetic variability observed within the Afrikaner cattle could be due to the present of strong selection and use of elite sires which is common among stud and commercial herds and small effective population size. This lower level should be noted in Afrikaner and step toward increasing diversity should be prioritized. This could include exchange of bulls from the different genetic pools. The negative correlation observed between allelic richness and expected heterozygosity in the Afrikaner cattle could be attributed to the processes that differential affect these two measures of diversity, such as bottleneck, selection and increased gene flow between populations within the Afrikaner (Comps et al., 2001).

Angus and Holstein cattle (H_e_ = 0.31) demonstrated the highest level of genetic variability compare to all other breeds. The highest genetic diversity observed in *Bos taurus* breeds were in agreement with the results of Lin et al. (2010) who reported highest genetic variability within *Bos taurus* compared to *Bos indicus* and also to Edea et al. (2013) who reported more genetic diversity in Hanwoo (H_e_ = 0.41) breed than in Ethiopia cattle breeds (between H_e_ = 0.37–0.38) based on SNP data. Heterozygosity values observed in this study were comparable to the previously reported heterozygosity among African (H_e_ = 0.25) and European (H_e_ = 0.30) cattle breeds using SNPs (Gautier et al., 2007). The levels of inbreeding observed in this study were lower across the breeds. However, it should be noted that this may not indicate the real status of inbreeding within these cattle breeds as allele frequencies may be poor estimate of inbreeding. Assessment of the inbreeding level should be done every 5 years to determine any unfavorable change in inbreeding levels, so that appropriate steps could be taken to prevent increases in inbreeding.

Analysis of molecular variance among indigenous and locally-developed breed revealed that about 90% of the genetic variation occurred within the populations. This was lower than the within-population genetic variation (99%) observed among Ethiopia populations by Edea et al. (2013). Combining all six breeds showed that 92% of total variation was within populations. This was higher than 81% observed among Ethiopia and Hanwoo cattle populations.

As expected genetic differentiation (*F_ST_*) among the indigenous and locally-developed breeds was lower than African-*Bos taurus* pairs, ranging from 4 to 8%. This was lower than 12% observed among West African cattle breeds by Gautier et al. (2007), but higher than 1% reported among Ethiopian cattle breeds (Edea et al., 2013). Among indigenous and locally-developed and *Bos taurus* cattle breeds genetic differentiation ranged between 8 and 15%; this was comparable to 15% reported between African and European breeds by Gautier et al. (2007) and 17% reported by Edea et al. (2013) among Ethiopia and Hanwoo cattle populations.

The average genetic distance between pairs of animals drawn from the same breeds ranged from 0.20 (Afrikaner) to 0.25 (Angus and Holstein). Average genetic distance between pairs of animal (0.21) was previously reported within 19 cattle breeds (Bovine HapMap Consortium, 2009). As expected average genetic distance between individuals drawn from different breed was higher than those drawn from within breeds, ranging from 0.23 (Nguni-Afrikaner) to 0.29 (Angus-Holstein).

Phylogenic analyses confirmed the closer relationship among indigenous and locally-developed breeds and clearly separated indigenous and locally-developed breeds from *Bos taurus* breeds; this was in agreement with the great divergence between African and European/British breeds observed by Gautier et al. (2007). It will be interesting to expand this breed level analysis in subsequent studies through the inclusion of all SA cattle breeds to better understand genetic relationship among SA cattle breeds.

Population structure analysis revealed some signals of admixture and genetic relationship between Afrikaner, Nguni and Drakensberger and Bonsmara. Nguni cattle shared some genetic links with the Afrikaner cattle, with about 8% of its genome derived from the Afrikaner cattle. This may reflect co-ancestry regarding the origin of these breeds as both these came from the same migration route into the Southern Africa (Scholtz et al., 2011). On the other hand, the Bonsmara cattle shared some genetic links with the Nguni cattle (3%) but only limited genetic links with Afrikaner cattle (0.5%); which was unexpected since the Bonsmara cattle was developed through crossbreeding of Afrikaner cattle with exotic breeds such as Hereford and Milk Shorthorn during the early sixties (Bonsma, 1980). However, it should be noted that when Afrikaner and Nguni cattle were brought to the Southern Africa by the Khoi-Khoi people, Afrikaner cattle migrated along the western side of Southern Africa whilst the Nguni cattle migrated along the eastern side of Southern African (Scholtz et al., 2011), and the Bonsmara cattle was developed in the eastern part of South Africa which predominantly consisted of the Nguni cattle. The observed low relationship between Bonsmara and Afrikaner may also be attributed to genetic drift or small sample size. The Drakensberger cattle was the most admixtured breed in this study with about 5% of its genome derived from the Nguni, Bonsmara and Angus and 3% from Afrikaner and Holstein; this was in agreement with the history of this breed which is believed to have unclear origin (Scholtz, 2010). Afrikaner cattle was the least admixed breed in this study, this was in agreement with the history of this breed as it was the first indigenous South African breed to form a breed society in 1912, thus this breeds may have been closed within the breeding society where only registered animals are allowed within the society. Limited genetic component was shared between indigenous *Bos taurus* breeds, this indicated distinct genetic resources in South African which should be utilization and conservation separately.

In general phylogenetic and population structure analysis revealed distinctiveness among South African (indigenous and locally-developed cattle breeds) and *Bos taurus* cattle breeds which is in agreement with their separate domestication and great time divergence (McKay et al., 2008). The presence of some admixture among South African cattle breeds was in accordance with previous results of genetic diversity studies among cattle breeds that are generally closer together geographically (McKay et al., 2008; Edea et al., 2013). This indicated that the genetic diversity of breeds is directly linked to the areas of origin, suggesting that breeds which have diverged more recently have a generally closer relationship than breeds which diverged long time ago (Maudet et al., 2002).

## Conclusion

This study revealed low to moderate genetic diversity within six cattle breeds in South Africa and showed a closer relationship among indigenous and locally-developed cattle breeds. Clear genetic divergence between South African (indigenous and locally-developed cattle breeds) and *Bos taurus* cattle breeds was observed which suggested distinct genetic resource in South Africa cattle breeds that should be proper utilization and conservation in order to cope with unpredictable future environments. Information generated from this study forms the basis for future management of these cattle breeds.

## Author contributions

Sithembile O. Makina collected the genetic materials, carried out the laboratory analyses, statistical analyses, interpretation of the data and drafted the manuscript. Azwihangwisi Maiwashe and Farai C. Muchadeyi assisted with the acquisition of funding. All authors participated in the design and coordination of the study. Azwihangwisi Maiwashe, Farai C. Muchadeyi, Este van Marle-Köster and Michael D. MacNeil revised the manuscript critically for important intellectual content. All authors read and approved the final manuscript.

### Conflict of interest statement

The authors declare that the research was conducted in the absence of any commercial or financial relationships that could be construed as a potential conflict of interest.
